# Reliable high-PAP-1-loaded polymeric micelles for cancer therapy: preparation, characterization, and evaluation of anti-tumor efficacy

**DOI:** 10.1080/10717544.2025.2490269

**Published:** 2025-04-10

**Authors:** Fang Ye, Qi Li, Longping Huang, Naikai Liao

**Affiliations:** a School of Basic Medical Sciences, Guangxi Medical University, Nanning, Guangxi, P. R. China; b Department of Pathology, The First Affiliated Hospital of Guangxi Medical University, Guangxi Medical University, Nanning, Guangxi, P. R. China; c Clinical Laboratory, The First Affiliated Hospital of Guangxi University of Chinese Medicine, Guangxi University of Chinese Medicine, Nanning, Guangxi, P.R. China; d Department of Urology, The First Affiliated Hospital of Guangxi Medical University, Guangxi Medical University, Nanning, Guangxi, P. R. China

**Keywords:** Mitochondrial potassium channel Kv1.3, PAP-1, pH-responsive, anti-tumor, polymeric micelles

## Abstract

The mitochondrial potassium channel Kv1.3 is a critical therapeutic target, as its blockade induces cancer cell apoptosis, highlighting its therapeutic potential. PAP-1, a potent and selective membrane-permeant Kv1.3 inhibitor, faces solubility challenges affecting its bioavailability and antitumor efficacy. To circumvent these challenges, we developed a tumor-targeting drug delivery system by encapsulating PAP-1 within pH-responsive mPEG-PAE polymeric micelles. These self-assembled micelles exhibited high entrapment efficiency (91.35%) and drug loading level (8.30%). As pH decreased, the micelles exhibited a significant increase in particle size and zeta potential, accompanied by a surge in PAP-1 release. Molecular simulations revealed that PAE’s tertiary amine protonation affected the self-assembly process, modifying hydrophobicity and resulting in larger, loosely packed particles. Furthermore, compared to free PAP-1 or PAP-1 combined with MDR inhibitors, PAP-1-loaded micelles significantly enhanced cytotoxicity and apoptosis induction in Jurkat and B16F10 cells, through mechanisms involving decreased mitochondrial membrane potential and elevated caspase-3 activity. *In vivo*, while free PAP-1 failed to reduce tumor size in a B16F10 melanoma mouse model, PAP-1-loaded micelles substantially suppressed tumors, reducing volume by up to 94.26%. Fluorescent-marked micelles effectively accumulated in mouse tumors, confirming their targeting efficiency. This strategy holds promise for significantly improving PAP-1’s antitumor efficacy in tumor therapy.

## 1. Introduction

As we all know, cancer is a deadly disease with significant morbidity and mortality rates (Bray *et al.* 2024). Chemotherapy stands as one of the most potent strategies for treating cancer. However, multidrug resistance (MDR) remains a significant hurdle in its clinical application, often leading to therapeutic failures (Hossain and Haldar 2023). A crucial aspect of this drug resistance is the downregulation of pro-apoptotic proteins, Bax and Bak, which are vital in regulating apoptosis (Jayappa *et al.* 2023). Remarkably, Leanza *et al.* discovered a fascinating finding: inhibiting mitochondrial Kv1.3 channels can trigger cancer cell apoptosis independently of Bax and Bak (Leanza *et al.* 2017).

Kv1.3 potassium channels, belonging to the Shaker potassium channel family (Varanita *et al.* 2023), have been observed to be expressed and functionally active not only in the plasma membrane but also in the inner mitochondrial membrane (mitoKv1.3) of lymphocytes (Szabò *et al.* 2005), hippocampal neurons (Bednarczyk *et al.* 2010), and astrocytes (WangTerrando and Orser 2020, Rangaraju *et al.* 2015). Plasma membrane-located Kv1.3 channels are essential for regulating cell proliferation (Navarro-Perez *et al.* 2023, Serrano-Albarras *et al.* 2018), whereas mitoKv1.3 channels are implicated in the process of apoptosis (Capera *et al.* 2022, Prosdocimi Checchetto and Leanza 2019). Changes in Kv1.3 expression have been documented in various cancers, including lymphoma (Hu *et al.* 2019), glioma (Bielanska *et al.* 2009), melanoma (Artym and Petty 2002), prostate (Fraser *et al.* 2003), breast (AbdulSanto and Hoosein 2003), gastric (Lan *et al.* 2005), pancreatic (Brevet *et al.* 2009), and colon cancers (Abdul and Hoosein 2002). Intriguingly, Bax physically interacts with mitoKv1.3 in apoptotic cells, significantly inhibiting Kv1.3 activity at nanomolar concentrations of Bax (Leanza *et al.* 2012, Szabó *et al.* 2008). This interaction initiates a series of apoptotic events, such as membrane potential shifts, reactive oxygen species (ROS) generation, and cytochrome c release (Kadow *et al.* 2022). Given cancer cells’ increased susceptibility to oxidative stress due to their altered redox balance, specifically targeting mitochondrial Kv1.3 with inhibitors appears to be a promising therapeutic approach for cancer treatment. This strategy could potentially overcome multidrug resistance challenges by mediating a Bax-independent apoptosis pathway.

Kv1.3 channel inhibitors encompass potent toxin inhibitors, such as Margatoxin (Bartok *et al.* 2014) and ShK (Varga *et al.* 2021, Ramesha *et al.* 2021), which function by occluding the channel. Nevertheless, these toxins cannot penetrate cells, prompting significant efforts to identify non-peptide small molecules capable of specifically inhibiting Kv1.3. Currently, numerous organic inhibitors, including Psora-4 (Venturini *et al.* 2017, Pereira *et al.* 2007), PAP-1 (Kundu-Raychaudhuri *et al.* 2014, Gubič *et al.* 2021), and clofazimine (Leanza *et al.* 2013) had been discovered. These inhibitors, unlike peptide inhibitors, can permeate biomembranes and operate by binding within the inner pore or at the interface between Kv1.3 subunits. Notably, PAP-1 (5-(4-phenoxybutoxy) psoralen) stands out as the most potent and selective membrane-permeant Kv1.3 inhibitor and effectively blocks the channel with an IC_50_ of 2 nM. Furthermore, PAP-1 demonstrates a remarkable selectivity for Kv1.3, being 23-fold more specific than for Kv1.5 and exhibiting a 33- to 125-fold selectivity over other Kv1-family channels. Additionally, it exhibits over 1,000 times lower affinity for HERG (Kv11.1) and other channels (Peruzzo *et al.* 2020). This high selectivity makes PAP-1 a promising therapeutic agent for targeting Kv1.3 specifically, with minimal off-target effects. PAP-1 is currently undergoing a phase Ib clinical trial for psoriasis but holds promise for treating other autoimmune diseases and tumors as well. Significantly, PAP-1 exhibited no signs of acute toxicity *in vitro* or *in vivo* and did not possess phototoxicity. Despite its potency and selectivity as a Kv1.3 inhibitor, PAP-1 demonstrated a poor anti-tumor effect due to its poor water solubility, low targeting capability, and susceptibility to efflux by multidrug resistance (MDR) pumps. According to Leanza *et al.*, PAP-1’s anti-tumor effect *in vivo* is not statistically significant compared to the untreated group. Given these findings, there is a pressing need to develop a new formulation for PAP-1 that addresses its current limitations, including improving its water solubility, enhancing its tumor-targeting capabilities, and increasing its resistance to efflux by MDR pumps.

The nano drug delivery system has become a vital tool for increasing drug water solubility, regulating release rates, boosting drug safety, and elevating its therapeutic effectiveness, with amphiphilic polymer micelles (PMs) standing out for their ability to self-assemble in water, forming a core/shell structure that offers exceptional biocompatibility, high drug loading capacity, and prolonged circulation time (Li *et al.* 2021). Lipophilic drugs can be encapsulated within these nano-sized micelles, thereby boosting their water solubility (Luo *et al.* 2023, Lv *et al.* 2021). The interest in micelles has escalated because of their unique capacity to internalize via endocytosis, efficiently avoiding ATP-dependent efflux pumps, specifically the P-glycoprotein (P-gp) efflux pumps, thus significantly reducing drug resistance, especially multidrug resistance (MDR) (Ghezzi *et al.* 2021). Additionally, recent research demonstrated that PEG can further elevate drug delivery efficiency by boosting cell membrane permeability via the suppression of P-gp efflux pumps (Akhtar *et al.* 2011). In addition, stimuli-responsive polymeric micelles, have garnered attention for their potential in controlled drug release within tumor tissue, triggered by factors like temperature, ultrasound, electrical/magnetic fields, or pH changes. (Qiu *et al.* 2021). Among these triggers, exploiting pH changes at the tumor site holds particular promise. This acidic environment provides an ideal precondition for a pH stimulus-response drug delivery system in cancer therapy. Such a system retains the drug at a normal physiological pH of 7.4 but triggers rapid release at the acidic pH of tumor sites (ranging from approximately 6.5 to 7.0) (Zhang *et al.* 2022). For instance, poly(β-amino ester) (PAE), a degradable cationic polymer containing tertiary amine groups, is often employed as the hydrophobic, pH-responsive core material for polymeric nanocarriers. This is primarily due to its tertiary amine groups, which, with a p*K*b of about 6.5, make it highly responsive to the acidic extracellular pH of tumors, thereby enabling precise and efficient cancer therapy. The association of polyethylene glycol (PEG) polymer as a hydrophilic shell to PAE is a common practice achieved through a Michael-type step polymerization. Ko *et al.* and Cheng *et al.* successfully employed mPEG-PAE to deliver doxorubicin (DOX) or a combination of ERD308 and Palbociclib (Pal) (ChengHu and Cheng 2023, Ko *et al.* 2007). These studies demonstrated excellent efficacy in tumor reduction and showed a good safety profile.

Therefore, the herein study proposes the development of PAP-1-loaded mPEG-PAE micelles (PAP-1 PMs), aiming to overcome PAP-1’s poor water solubility and ensure that the nanoparticles specifically respond to the low extracellular pH of tumors for precise targeted delivery. The physicochemical properties of the NPs, such as particle sizes, zeta potential, and *in vitro* release profiling, were also examined using a variety of experimental techniques. Molecular dynamics simulations were employed to elucidate the molecular assembly mechanisms. For the first time, we found that encapsulating PAP-1 within micelles aided in overcoming its poor water solubility, thereby enhancing antitumor activity both *in vitro* and *in vivo* and improving tumor accumulation.

## 2. Materials and methods

### 2.1. Materials and animals

The mPEG-PAE was synthesized in our laboratory, with MPEG-propylene supplied by Ruixi Biological Technology Co., LTD (Xi’an, China). 1,6-bis (acryloyloxy) hexane and 1,3-bis (4-piperidine) propane were acquired from TCI Co., LTD (Shanghai, China). PAP-1, staurosporine, probenecid, and cyclosporin were bought from MedChemExpress Co., LTD. (Monmouth Junction, New Jersey, USA). Tween-20 and dimethyl formamide were supplied from Fuyu Chemical Co. LTD (Tianjin, China). Dimethyl sulfoxide and PBS were bought from Solarbio. Co. LTD (Beijing, China). Trichloromethane was provided by Kelong Chemical Co. LTD (Chengdu, China). CDCl_3_-*d* was derived from Macklin Biochemical Co., LTD (Shanghai, China). Methanol and acetonitrile were provided by Thermo Fisher Scientific Inc. (Waltham, Massachusetts, USA). DiD was bought from Biolite Corporation (Xian, China). Cell counting kit-8 was purchased by Labgic Technology Co. LTD. Annexin V-FITC/PI Apoptosis Kit was purchased from MultiSciences (Lianke) Biotech Co., LTD (Hangzhou, Zhejiang, China). Enhanced mitochondrial membrane potential assay kit with JC-1, Caspase-3 activity and apoptosis detection kit were derived from Beyotime Biotech Inc. (Shanghai, China). Jurkat cells (Catalog #: TCHU123) and B16F10 cells (Catalog #: TCM36) were acquired from the Cell Bank of the Chinese Academy of Sciences (Shanghai, China) and cultured in RPMI-1640 medium (Gibco, USA) and Dulbecco’s Modified Eagle’s Medium (DMEM, Gibco, USA) supplemented with 10% fetal bovine serum (FBS, Biological Industries LTD., Haemek, Israel). Avertin was obtained from MeilunBio LTD (Dalian, China). The protocol for animal experiments was approved by the Animal Experimentation Ethics Committee of Guangxi Medical University. Female C57BL/6J mice (4 weeks) were purchased and maintained in the Experimental Animal Center of Guangxi Medical University. All animal experiments were approved by the Animal Ethical and Welfare Committee of Guangxi Medical University.

### 2.2. Synthesis and characterization of mPEG-PAE polymers

The synthesis route for mPEG-PAE is illustrated in Figure S1. MPEG-propylene (1 g) was dissolved in 10 mL trichloromethane, and then 1,6-bis (acryloyloxy) hexane (10.0 eq.) and 1,3-bis (4-piperidine) propane (11.0 eq.) were added. The mixture was stirred at 55 °C for 48 h. Subsequently, the reaction solution was evaporated under reduced pressure and precipitated in chilled ethylether. The final product was filtered and vacuum-dried at room temperature.

The number average molecular weight (M_n_) and polydispersity index (M_n_/M_w_) were determined by gel permeation chromatography (GPC), conducted by a Nexera GPC system (Shimadzu Corporation, Kyoto, Japan). The GPC system was equipped with two Waters styragel columns (HR3 and HR4) in series, an LC-20AD pump, and a RID-20AD refractive detector. The analysis was performed using dichloromethane as the eluent, maintained at a flow rate of 1 mL/min and an operating temperature of 30 °C.

The polymer structure was characterized by proton nuclear magnetic resonance (^1^H-NMR) spectroscopy, conducted on a 400 MHz spectrometer (Bruker ACANCE III HD 400, Switzerland) at 25 °C. Deuterated chloroform (CDCl_3_-*d*) and tetramethylsilane were used as solvents and an internal standard, respectively.

### 2.3. Preparation and characterization of PAP-1 polymeric micelles

The PAP-1-loaded mPEG-PAE micelles (PAP-1 PMs) were prepared following the ultrasonic emulsification-solvent evaporation method. Initially, a precise quantity of PAP-1 (1 mg) and mPEG-PAE (10 mg) copolymers were dissolved in 1 mL of trichloromethane to obtain the organic phase. Subsequently, the organic phase was injected into the aqueous phase (distilled water adjusted to pH7.4) in a defined ratio of 1:3. The mixture was then processed using an ultrasonic homogenizer (JY96-IIN, Huxi Industrial Co. Ltd, Shanghai, China) for emulsification. The ultrasonic treatment was performed in cycles of 4 seconds of sonication followed by 2 seconds of pause, with a total sonication time of 1 minute, and the ultrasonic power was set at 40 watts.

Following ultrasonic emulsification, the resulting emulsion was subjected to rotary evaporation under vacuum conditions to effectively remove the organic solvent completely. Consequently, the PAP-1 and co-polymers spontaneously assembled to form a core-shell structure that wrapped PAP-1 inside the hydrophobic core. Moreover, the unencapsulated drug was removed by ultrafiltration to obtain the PAP-1 PMs. The DiD-marked polymeric micelles (DiD-M PMs) and the blank polymeric micelles (blank PMs) were also prepared according to the same protocol.

The characterization of the obtained polymeric micelles, including the average particle size, zeta-potential, and polydispersion index (PDI), were determined using a dynamic light scattering (DLS) method with a BI-200SM (Brookhaven Instruments Co. LTD., NY, USA). To visualize the morphology of the PAP-1 micelles, transmission electron microscopy (TEM, H-7650, Hitachi Nake High-Tech Corporation, Tokyo, Japan) was employed after negatively staining the samples with phosphotungstic acid for 10 min and drying in air, operating at 80 kV. X-ray diffraction (XRD) spectra of PAP-1, mPEG-PAE polymer, the physical mixture of PAP-1 and mPEG-PAE, and PAP-1 PMs were determined using an X-ray diffractometer (Panalytical X’Pert^3^ Powder, PANalytical, Almelo, The Netherlands). The diffraction angle was increased from 5° to 40°.

### 2.4. HPLC analysis for the quantification of PAP-1

The content of PAP-1 encapsulated within polymeric micelles was quantitatively analyzed using high-performance liquid chromatography (HPLC) (LC-16, Shimadzu Corporation, Kyoto, Japan). The polymeric micelles were dissolved in dimethyl formamide (DMF) under vigorous vortexing for 2 min and then filtered using a 0.22 μm membrane filter. The supernatant was collected for HPLC analysis. Standards and samples were injected in 20 μL aliquots into a reversed-phase C18 column (RD-C18, 250 × 4.6 mm; 5 μm particle size), employing a detection wavelength of 310 nm. The mobile phase consisted of a mixture of methanol and water (90:10, v/v) with a total flow rate of 1.0 mL/min. The entrapment efficiency (EE) and drug loading (DL) were calculated according to the following equations (Eqs. (1) and (2)):

(1) $$\text{EE}(\text{\%})\text{=}\frac{\text{Weight of loaded drug}}{\text{Weight}\;o\text{f}\;\text{drug in feed}}\times \text{100\%}$$

(2) $$\text{DL}(\text{\%})\text{=}\frac{\text{Weight of loaded drug}}{\text{Weight of drug loaded micelle}}\times \text{100\%}$$

### 2.5. Potentiometric titration and pH sensitivity of the micelles

MPEG-PAE was dissolved in HCl-containing distilled water, yielding a polymer solution (1 mg/mL, pH 3.0). This solution was then titrated with NaOH (0.1 mol/L) in 10 μL increments. The pH values were continuously monitored using a potentiometric titrator (PHS-3E, INASE Scientific Instrument Co., LTD, Shanghai, China) at 25 °C. Furthermore, the particle size and zeta potential of PAP-1 PMs were determined across various pH levels.

### 2.6. In vitro release of PAP-1 from MPEG-PAE micelles

The *in vitro* drug release profiles were investigated using a dialysis bag (MWCO 3,500 Da) at 37 °C. To maintain sink conditions, the drug release test was performed at a low drug concentration. In brief, 1 mL of PAP-1 PMs (100 μg/mL of PAP-1) was placed in a dialysis bag which was then submerged in 10 mL of PBS solution adjusted to pH 6.5 or 7.4, supplemented with 0.5% (w/v) Tween-80 served as the releasing medium. The beaker containing the above-mentioned dialysis bag and releasing medium was positioned in a 37 °C-water bath with gentle agitation. At predefined intervals (1, 3, 12, 24, and 48 hours), 10 mL of release medium was withdrawn and replaced with an equivalent volume of fresh medium. Subsequently, the PAP-1 concentration in the medium was determined through HPLC measurements.

### 2.7. Molecular dynamics simulation

In this study, we employed molecular dynamics simulations to investigate the driving interactions involved in the assembly process. The monomer molecules PAP-1 and mPEG-PAE were initially modeled using ChemBioDraw, and the MMFF94 force field was applied for optimization. Subsequently, the Packmol program (Martínez *et al.* 2009) was utilized to construct three distinct mixture systems in a predefined ratio, specifically composed of PAP-1 and mPEG-PAE in a 20:10 molar ratio. The three mixture systems were designated as follows: 1) “PAP-1 PMs-MinP,” where the tertiary amine moieties of PAE were minimally protonated; 2) “PAP-1 PMs-PartP,” consisting of partially protonated PAE segments; and 3) “PAP-1 PMs-FullP,” with fully protonated tertiary diamine moieties of PAE. To characterize the interactions within the systems, the GAFF force field (Wang *et al.* 2004) was employed for parameterization. The charge distribution of each atom was determined using the BCC method (Jakalian *et al.* 2002). Molecular dynamics simulations were performed using the GROMACS2018.4 software package (Van Der Spoel *et al.* 2005). After energy minimization, the systems were solvated in a TIP3P water box, and chloride ions were added to maintain a net charge of zero. During the equilibration phase, 50,000 steps of energy minimization were conducted. The NPT ensemble conditions were employed for the molecular dynamics simulations, and Newton’s equations of motion were solved with a time step of 2 fs. The V-rescale temperature coupling method was applied to maintain the simulation temperature at 300 K, while the Berendsen method controlled the pressure at 1 bar. Electrostatic interactions were handled using the Particle Mesh Ewald (PME) method. To analyze the structural characteristics and dynamics of the system, various metrics were evaluated. The radius of gyration (Rg) was calculated to assess the compactness of the structure. The solvent accessible surface area (SASA) was monitored to observe variations in the composite’s solvent-accessible surface area throughout the simulation. Root mean square deviation (RMSD) was calculated to assess the conformational stability of the system and detect significant structural changes over time. By saving configurations at regular intervals of 10 ps, we obtained trajectory data for subsequent analysis. These metrics, including RMSD, SASA, and Rg, provided valuable insights into the structural characteristics and dynamics of the system during the assembly process.

### 2.8. Cytotoxicity assays

The *in vitro* cytotoxicity in Jurkat and B16F10 cells was tested using a standard CCK-8 assay. Jurkat and B16F10 cells were grown in RPMI-1640 and DMEM medium, respectively. The medium was supplemented with 10% fetal bovine serum (FBS). All cells were placed in an incubator at 37 °C under a 5% CO_2_ atmosphere. Cells at the logarithmic phase were seeded in the 96-well plates at the density of 5 × 10^3^ to 1.5 × 10^4^ cells per well depending on the cell line. Six wells were designated for each experimental condition. Cells were treated with various concentrations of PAP-1, PAP-1 combined with MDR inhibitors (MDRi, including 4 μmol/L Cyclosporin H (CSH) and 100 μmol/L probenecid (Prob.), blank micelles, PAP-1 PMs, was added and treated cells for 24 hours. Following treatment, the culture medium was then replaced by the fresh medium containing CCK-8 (5 mg/mL) according to the procedure suggested in the manufacturer’s protocol. After a 3-hour incubation period at 37 °C for color development, the absorbance of each well was determined at 450 nm using a microplate reader (Gary 3500, Agilent Technologies Inc. Santa Clara, USA).

The relative cell viability (%) was calculated using the following equation (Eq. (3)):

(3) $$\text{Cell viability}(\text{\%})\text{=}\frac{A_{\text{sample}}‐A_{\text{blank}}}{A_{\text{control}}‐A_{\text{blank}}}\times \text{100\%}$$

A_control_ and A_sample_ are absorbance at 450 nm in the absence and in the presence of sample treatment, respectively. A_blank_ is the absorbance at 450 nm without cells.

### 2.9. Apoptosis assay

An Annexin V-FITC/PI assay was performed to analyze cell apoptosis in Jurkat and B16F10 cells. Depending on the cell line, the cells were seeded at a density of 8 × 10^4^ cells/well in either 12-well plates or 24-well plates. The cells were treated with various concentrations of PAP-1, PAP-1 combined with MDRi, blank micelles, and PAP-1 PMs for 24 h. After treatment, the cells were harvested and washed three times with PBS. Subsequently, the cells were treated in accordance with the procedure suggested in the Annexin V-FITC/PI apoptosis kit (Multi-Sciences, Hangzhou, China). Apoptosis was measured using a flow cytometer (Guava EasyCyte, EMD Millipore Corporation, Billerica, USA) and the data were analyzed using FlowJo_v10.6.2 software.

Utilizing caspase-3/Annexin V dual staining assays, we sought to investigate the role of caspases in apoptosis induced by diverse formulations. In this study, Jurkat cells, seeded at a density of 8 × 10^5^ per well, were exposed to various PAP-1 treatment formulations at concentrations equivalent to 50 μM or blank micelles for a duration of 24 hours. The control group refers to the group that did not receive treatment drugs. Following incubation, the cells were centrifuged and resuspended in PBS. The subsequent staining procedure, involving dual red-green fluorescence, adhered strictly to the guidelines provided by the manufacturer of the Caspase-3 Activity and Apoptosis Detection Kit. Caspase-3, a critical enzyme involved in apoptosis, was detected using GreenNuc^™^ caspase-3 substrate, a fluorescent-labeled polypeptide that is specifically recognized by caspase 3/7, resulting in the emission of a bright green fluorescence. Moreover, the exposure of phosphatidylserine on the cell surface, a distinctive feature of apoptosis, was identified through Annexin V-mCherry staining. The entire process culminated in the examination of the stained cells under a confocal laser scanning microscope (CLSM, LSM800, Zeiss, Göttingen, Germany).

### 2.10. Measurement of mitochondrial membrane potential JC-1

Jurkat cells were treated with PAP-1 alone, a combination of PAP-1 and MDRi, blank PMs, and PAP-1 PMs at a concentration equivalent of 50 μM of PAP-1 for 2 or 4 hours at 37 °C. The control group was not administered with the treatment drug. Following the treatments, the Jurkat cells were prepared for staining with mitochondrial membrane potential JC-1 dye according to the manufacturer’s protocol. The Jurkat cells were washed twice with PBS, and then 1 μM of the potential-sensitive dye JC-1 was added before being returned to the incubator. Following a 20-minute incubation period, the Jurkat underwent three PBS washes, and finally, images were captured using Confocal Laser Scanning Microscopy (CLSM). In healthy cells, JC-1 dye preferentially accumulates in the mitochondria due to the electrochemical gradient across the mitochondrial membrane (ΔΨ_m_), leading to aggregation and emission of red fluorescence. However, in the cytosol, JC-1 remains monomeric, emitting green fluorescence. Disruptions in ΔΨ_m_, a crucial prelude to cytochrome c translocation and apoptosis initiation, are indicated by a reduction in red fluorescence coupled with an increase in green fluorescence.

### 2.11. In vivo anti-tumor therapy and histological analysis

To evaluate the anti-tumor activities in vivo, a B16F10 mouse model of melanoma was established. This study involving animals had been conducted and reported in accordance with the ARRIVE guidelines (https://arriveguidelines.org/), aimed at providing comprehensive information and promoting rigorous design and reporting of research. This study utilized 76 female C57BL/6J mice for a pilot experiment to evaluate tolerance to various doses of PAP-1 and PAP-1 loaded micelles and assess anti-tumor activities and distribution of PAP-1-loaded polymeric micelles. To create this model, B16F10 cells were initially cultured in T25 flasks until they achieved 80% confluence. After reaching this confluence, the cells were harvested and thoroughly washed twice with phosphate-buffered saline (PBS). Subsequently, a cell suspension containing 5 × 10^4^ B16F10 cells was prepared and injected subcutaneously into the right flank of C57BL/6 mice. The B16F10 mouse model of melanoma has gained widespread use in research, as noted in Peruzzo et al. (2020). Leanza *et al.* found that administering PAP-1 alone at a dosage of 7 μg/g to mice with melanoma tumors did not effectively reduce their tumor volume. Consequently, we attempted to increase the PAP-1 dosage by two-fold in our preliminary experiments. However, mice given this higher dosage displayed severe adverse reactions, including trembling and convulsions, which may possibly have been caused by toxic effects related to the overdose of PAP-1 or its solubilizer. In contrast, a two-fold higher dose of PAP-1 PMs (14 μg/g equivalent to PAP-1) caused no adverse effects. Therefore, all experimental mice were randomly divided into six groups (*n* = 6) and administered the following treatment: control (saline), PAP-1 ((7 μg/g body weight, gbw)), low dose blank PMs, high dose blank PMs, low dose PAP-1 PMs (7 μg/g equivalent to PAP-1), high dose PAP-1 PMs (14 μg/g equivalent to PAP-1).

The formulations were intraperitoneally administrated on the 5th day following tumor inoculation, with subsequent administrations on days 7, 9, and 11. Tumor volumes and body weights were monitored and recorded during the experiment. Tumor dimensions, specifically lengths (L) and widths (W) were measured using calipers, and tumor volumes were calculated using the: V = (L × W^2^)/2, where W is shorter than L. After 17 days, the tumors were excised and weighted to determine the tumor burden using the formula: tumor burden (%) = (W_tumor_/W_mice_) × 100. Moreover, main organs such as the heart, kidney, lung, spleen, liver, small intestine, and brain were dissected from the sacrificed mice were dissected. These organs underwent fixation in 4% formaldehyde, embedding in paraffin, and sectioning into 5 μm slices for hematoxylin and eosin (H&E) staining to facilitate histological evaluation. The H&E-stained tissues were imaged by microscopy (BX53, Olympus Corporation, Tokyo, Japan). All animal experiments were performed according to the Guide for Care and Use of Laboratory Animals of Guangxi Medical University.

### 2.12. In vivo biodistribution

C57BL/6 mice bearing B16F10 tumors were utilized to investigate the biodistribution of polymeric micelles through fluorescence imaging. Once the B16F10 tumor attained a volume of approximately 300 mm^3^, the mice were randomly divided into three groups and received intraperitoneal administrations of saline, a free DiD solution (1 μg/g body weight, gbw), and DiD-marked micelles (at a dose equivalent to 1 μg/g DiD), respectively. Fluorescent images were captured at predefined intervals (0, 2, 4, 6, 8, and 24 h) using an *in vivo* optical imaging system (IVIS, Analytik-Jena GmbH, Jena, Germany) spectrum, with three mice per group. Mice were anesthetized by intraperitoneal injection of a 20 mg/mL avertin solution. Following the 24-hour post-injection, the mice were euthanized, and key tissues including the heart, kidney, lung, spleen, liver, small intestine, brain, and tumor were excised for *ex vivo* imaging.

### 2.13. Statistics analysis

The experimental data were presented as average values, denoted as mean ± SD (standard deviation), and were based on three independent measurements, unless otherwise indicated. Statistical significance was determined by a one-way ANOVA test (GraphPad Prism, Version 8.3.0). Significance levels were indicated as follows: *P* < 0.05 indicated statistical significance (*), *P* < 0.01 signified high significance (**), and *P* < 0.001 represented remarkable significance (***).

## 3. Results and discussion

### 3.1. Synthesis and characterization of MPEG-PAE copolymers

As shown in Figure S1, MPEG-PAE was synthesized via a Michael-type polymerization reaction. The 1H-NMR spectrum confirming the chemical structure of the obtained MPEG-PAE (Figure S2) provided evidence for the successful synthesis of the amphiphilic block copolymer mPEG-PAE. Gel permeation chromatography (GPC) measurements indicated an average molecular weight (Mn) of 15.3 kDa for the synthesized mPEG-PAE. Additionally, a polydispersity index of approximately 1.87 suggested a narrow molecular weight distribution for the synthesized polymer.

### 3.2. Characterization of PAP-1-loaded polymeric micelles

**Figure 1.**
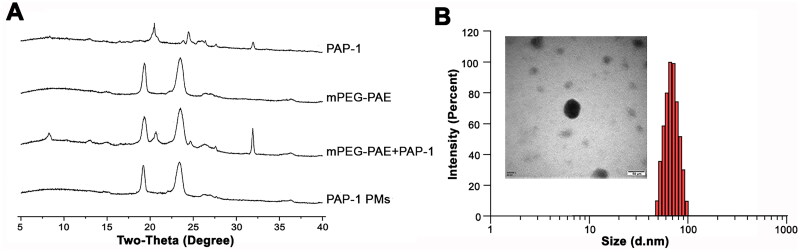
The XRD spectrum, size distribution, and TEM image of PAP-1 PMs. (A). XRD patterns of PAP-1, blank mPEG-PAE micelles, physical mixture of mPEG-PAE + PAP-1, and PAP-1 PMs. (B). Size distribution and transmission electron microscopy micrograph of PAP-1-loaded PMs. Scale bar = 50 nm.

X-ray diffraction (XRD) analysis, presented in Figure 1(A), revealed that the diffraction peaks of PAP-1 almost disappeared in the PAP-1 loaded PMs compared to the physical mixture of PAP-1 and mPEG-PAE, suggesting successful encapsulation of PAP-1 within the mPEG-PAE micelles. PAP-1-loaded NPs were characterized using dynamic light scattering (DLS), revealing an average diameter of 64.47 ± 0.37 nm and a zeta potential of −0.81 ± 0.57 mV for the PAP-1 PMs. The particles exhibited a polydispersity index (PDI) of 0.12 ± 0.01 (PDI < 0.2), indicating a narrow size distribution and homogenous dispersion (Figure 1B). The entrapment efficiency and drug loading of PAP-1 within the polymer were determined to be 91.35 ± 1.13% and 8.30 ± 0.10%, respectively (Table 1). TEM imaging further confirmed the spherical morphology of the PAP-1-loaded PMs (Figure 1B), with a light periphery surrounding a dark center, indicating a core-shell structure.

**Table 1.** Particle size, polydispersity index, zeta potential, entrapment efficiency, and drug loading level of the PAP-1 PMs.

Sample	Particle size (mean ± S.D.*)(nm)	Polydispersity (mean ± S.D.*)	Zeta potential (mV) (mean ± S.D.*)	Entrapment efficiency (%) (mean ± S.D.*)	Drug loading (%) (mean ± S.D.*)
PAP-1-mPEG-PAE	64.47 ± 0.37	0.12 ± 0.01	−0.81 ± 0.57	91.35 ± 1.13%	8.30 ± 0.10%
n = 3					
S.D.: Standard deviation					

### 3.3. pH-sensitivity evaluation of the polymeric micelles

**Figure 2.**
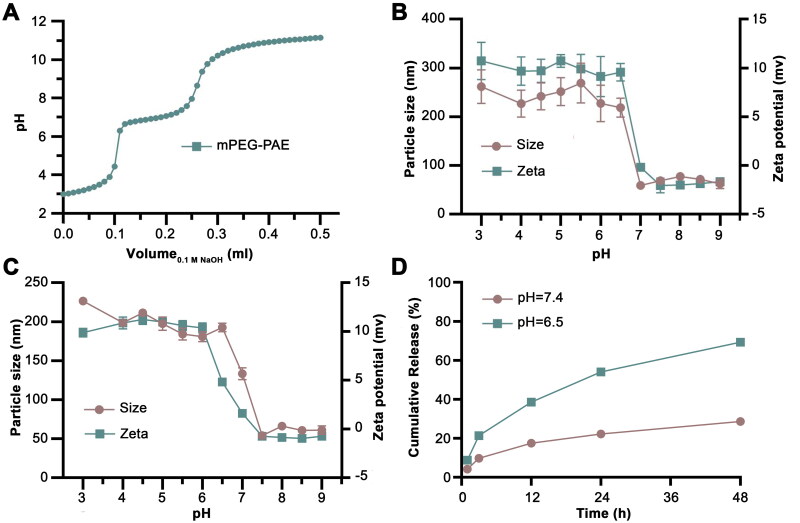
The pH responsiveness of the mPEG-PAE polymeric micelles. (A). The potentiometric titration of mPEG-PAE dependent on the different pH values. (B). Particle size and zeta potential of blank mPEG-PAE micelles. (C). Particle size and zeta potential of PAP-1 PMs. (D). *In vitro* release profiles of PAP-1 from PAP-1 PMs under pH conditions of 7.4 and 6.5.

The pH-sensitivity of the mPEG-PAE was evaluated by an acid-base titration. As depicted in Figure 2(A), upon initiating the addition of NaOH solution, the pH value gradually increased at first and then accelerated until reaching a plateau in the range of 6.3-7.5, which was attributed to the ionization of tertiary amine groups of PAE. With the further addition of NaOH, the pH rose sharply again. The results demonstrated that the pH sensitivity range of the tertiary amine group of MPEG-PAE was between 6.3–7.5, indicating the tertiary amine group of the PAE segment could protonate within this pH range. To further investigate the pH-sensitivity of the micelles, the particle size and zeta potential of blank micelles were detected under various pH conditions. Our results, presented in Figure 2(B), revealed that the particle sizes of the blank PM remained relatively consistence (approximately 60 nm) as the pH value decreased from 9.0 to 7.0. The consistency may be attributed to the fact that the tertiary amine moieties of PAE were barely protonated, which resulted in a compact particle structure. However, as the pH decreased to 6.5 and below, the PM sizes increased sharply to around 220 nm, followed by a slight increase, stabilizing at around 260 nm. This significant increase could be explained by the protonation of the tertiary amine groups of PAE, which led to their hydrophobic to hydrophilic transition. As a result, the micelle shifted from a compact to a swollen state, significantly increasing their size. In terms of zeta potential, our results demonstrated that the zeta potential of the particulate matter (PMs) slightly increased from −1.68 mV to −0.2 mV as the pH value decreased from 9.0 to 7.0. However, a notable surge in zeta potential was observed at a pH of 6.5, where it rapidly rose to 9.6 mV. This increase in positive charge enhanced the repulsive forces among the out-stretched hydrophilic chains of the PAE segment, prompting further loosening of the micelle structure and a subsequent increase in particle size. As the pH continued to decline, the zeta potential almost reached a plateau, reflecting the complete protonation of tertiary amine groups.

The response of PAP-1 PMs to pH changes mirrored that of blank micelles. As depicted in Figure 2(C), the micelles maintained a relatively constant particle size at pH levels from 9.0 to 7.5. However, a notable increase in particle size was observed as the pH decreased to 7.0, swelling from 54 nm to 133 nm. This enlargement was triggered by the partial protonation of the PAE segment, which led to a looser and expanded structure. Below a pH of 6.5, the particle size underwent a drastic surge, peaking at approximately 190 nm. Within the pH range of 6.0–3.0, the particle size experienced a slight increase, stabilizing at around 210 nm. Additionally, the zeta potential of the PAP-1 PMs demonstrated pH sensitivity. It remained steady at approximately −1.0 mV as the pH gradually decreased from 9.0 to 7.5, but underwent a significant shift from negative to positive, reaching 4.83 mV, when the pH dropped to 6.5. As the pH further decreased within the range of 6.0–3.0, the zeta potential continued to rise, plateauing at approximately 10 mV due to the complete protonation of the tertiary diamine groups.

### 3.4. In vitro release of PAP-1 from micelles

The PAP-1 PMs exhibited pH-responsive drug release, as confirmed by the HPLC method. As depicted in Figure 2(D), PAP-1 release rates were markedly accelerated in acidic conditions (PBS, pH 6.5) compared to physiological conditions (PBS, pH 7.4). At pH 7.4, the PAP-1 PMs maintain a compact structure, slowing PAP-1 release to just 17.51 ± 0.17% after 12 hours and 28.67 ± 0.30% after 48 hours, indicating effective encapsulation within the micelles’ dense conformation and prevention of burst release. However, at pH 6.5, the PAP-1 release rate was enhanced significantly, and the cumulative release was 38.53 ± 0.74% after 12 hours and 69.31 ± 0.50% after 48 hours, highlighting the micelle system’s pH-dependent release property. This could be explained by the protonation of tertiary amine groups in the PAE moieties at lower pH, causing a micelle structure loosening. Additionally, lower pH augments the ionization of PAE moieties, elevating surface charge density and causing electrostatic repulsion among PAE segments, relaxing the micelle structure, and hastening PAP-1 release. The aforementioned characterization results also confirmed that the particle size of PMs increased as the pH decreased. In brief, the polymeric micelle effectively compressed and protected PAP-1 at pH 7.4, while significantly accelerating its release at 6.5, exhibiting pH-sensitive controlled release behavior, which is highly valuable in weakly acidic tumor conditions where PAE ionization facilitates micelle swelling and accelerates PAP-1 release.

### 3.5. Molecular dynamics simulations

**Figure 3.**
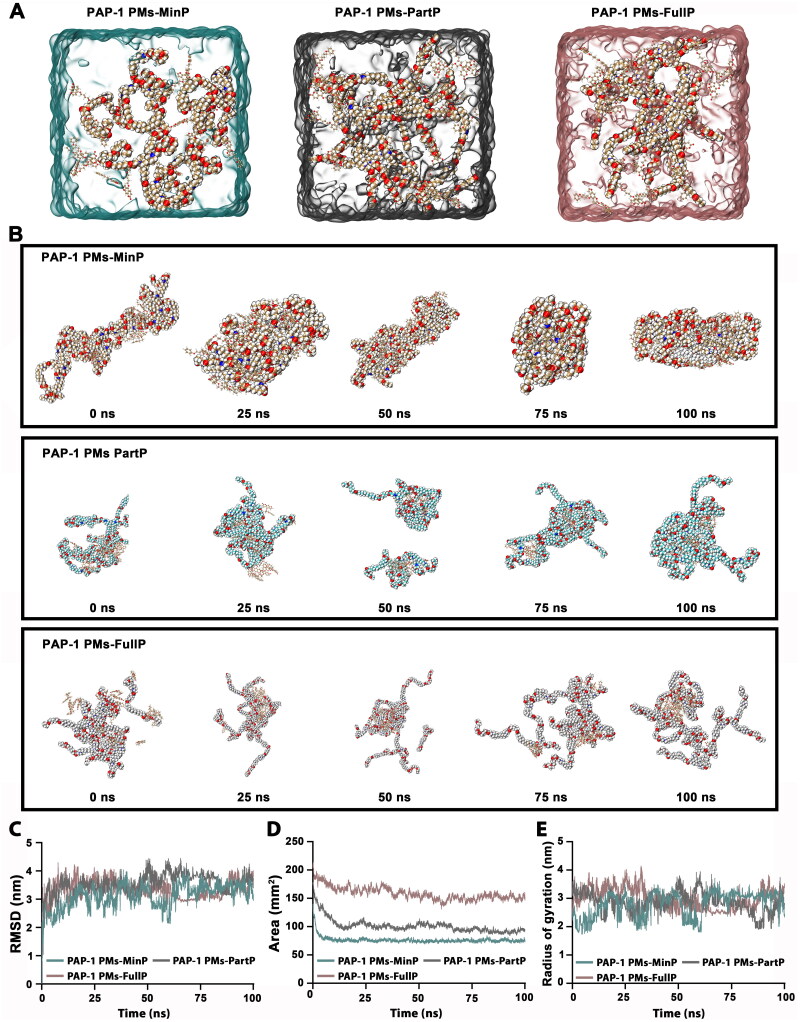
The Assembly process of the PAP-1-loaded mPEG-PAE micelles, encompassing various protonation states of the PAE tertiary amine moieties: minimally protonated (designated as PAP-1 PMs-MinP), partially protonated (designated as PAP-1 PMs-PartP), and fully protonated (designated as PAP-1 PMs-FullP). (a). The images of the three nano assembly systems where PAP-1 and mPEG-PAE were simultaneously placed, with the PAE moieties of mPEG-PAE exhibiting three protonation states: fully protonated, partially protonated, and fully protonated. Each system was placed in an individual TIP3P water box. (B). The assembly process of PAP-1 and the various states of mPEG-PAE (the upper: minimal protonation; the Middle: partial protonation; the bottom: full protonation) observed over a time span of 0 to 100 ns. (C). RMSD. (D). SASA. (E). Radius of gyration.

Furthermore, the present study utilized molecular dynamics simulations to investigate the self-assembly mechanisms of PAP-1 and mPEG-PAE in different protonation states. Three distinct mixture systems were studied, including PAP-1 combined with minimally, partially, and fully protonated mPEG-PAE. The molar proportions of the components and added chloride ions to maintain ionic equilibrium were determined as detailed in Table S1. Each system was placed in a separate TIP3P water box (Figure 3A). The molecular dynamics simulation results, depicted in Figure 3(B), revealed distinct self-assembly behaviors among the systems. Within approximately 25 nanoseconds (ns), PAP-1 and minimally protonated mPEG-PAE spontaneously formed a stable nanostructure, with the hydrophilic PEG segments facing outward. In contrast, the systems with partially or fully protonated mPEG-PAE exhibited significantly sparser configurations due to strong repulsive charge interactions between the positively charged chains. This prevented tight packing and the formation of compact structures. As the simulation progressed, some groups containing partially and fully protonated mPEG-PAE dissociated, while the system with minimally protonated mPEG-PAE continued to assemble into a more compact cluster. At the end of the 100 ns simulation, it was evident that PAP-1 and minimally protonated mPEG-PAE exhibited superior self-assembling capability compared to the systems with partially or fully protonated mPEG-PAE. These findings underscore the critical role of protonation in governing the self-assembly process.

Analysis of the root-mean-squared deviation (RMSD) curve revealed that the system of PAP-1 with unprotonated mPEG-PAE reached an RMSD value of approximately 3.15 nm by the end of the 100 ns simulation. In contrast, the systems with partially and fully protonated PAE segments exhibited RMSD values of 3.66 nm and 3.88 nm, respectively. This indicates a greater degree of structural flexibility in the systems with protonated PAE segments compared to the unprotonated system (Figure 3C). Furthermore, the solvent-accessible surface areas (SASA) of the three systems decreased after the initial 25 ns, confirming the self-assembly trend. The SASA values for the systems with minimally, partially, and fully protonated PAE segments were 74.44 mm^2^, 94.32 mm^2^, and 152.35 mm^2^, respectively. This indicates that the systems with protonated PAE segments exhibited more swollen features compared to the system with minimally protonated PAE (Figure 3D). Additionally, the Rg values, a parameter that reveals the compactness of the structure, were 2.38 nm, 2.78 nm, and 3.39 nm for the systems with minimally, partially, and fully protonated PAE segments, respectively (Figure 3E), at the end of the 100 ns simulation, indicating that the system with minimally protonated PAE segments was the most compact. Overall, our simulation studies underscored the significance of protonation in the self-assembly process, validating our experimental findings that protonation at lower pH levels modified the hydrophobicity, leading to swollen and loosely structured particles with varying sizes. These observations established a basis for targeting the PAP-1 release behavior of our polymeric micelles specific to the acidic extracellular pH of tumors.

### 3.6. Cell cytotoxicity

**Figure 4.**
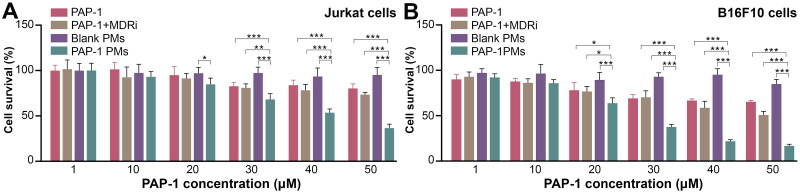
Cytotoxic effect of various PAP-1 formulations on jurkat and B16F10 cells. Cell viability was measured by CCK-8 assay. (A). Cell viability assessment of jurkat cells treated by blank micelles (a), or free PAP-1, free PAP-1 combined with multidrug resistance pump inhibitors (MDRi), and PAP-1 PMs. Cells were treated for 24 hours with diverse PAP-1 formulations, ensuring final PAP-1 concentrations of 1, 10, 20, 30, 40, and 50 μmol/L. The MDRi solution was prepared with a concentration of 4 μmol/L CSH and 100 μmol/L prob. After incubating with CCK-8 solution for 3 hours, absorbance values at 450 nm were measured using a microplate reader to calculate cell viability. (B). B16F10 cells underwent the same treatments as jurkat cells for 24 hours. Data were expressed as mean ± standard deviation (*n* = 6). (*) *p* < 0.05, (**) *p* < 0.01, (***) *p* < 0.001.

The cytotoxic effects of various treatments, including PAP-1 alone, PAP-1 combined with multidrug resistance pump inhibitors (MDRi), blank micelles, PAP-1-loaded MPEG-PAE micelles were investigated on Jurkat cells and B16F10 cells utilizing the CCK-8 method. As reported by Leanza *et al.*, PAP-1 can be extruded from tumor cells by multidrug resistance (MDR) pumps, a process that can be inhibited by MDR inhibitors (MDRi) to augment PAP-1’s intracellular effects. Thus, we conducted a comparative analysis of the cytotoxic effects of PAP-1 PMs (PAP-1 PMs) versus PAP-1 alone and PAP-1 in conjunction with MDR pump inhibitors, specifically 4 μmol/L Cyclosporin H (CSH) and 100 μmol/L probenecid (Prob.). In Jurkat cells, PAP-1 PMs exhibited stronger cytotoxicity compared to PAP-1 alone and PAP-1 combined with MDRi at a concentration of 30 µmol/L. However, the numerical difference gap was not large, with cell viabilities of 82.74%, 80.88%, and 68.13% for PAP-1 and PAP-1 + MDRi, and PAP-1 loaded PMs treatment groups, respectively. At 50 µmol/L, the viability of Jurkat cells treated with PAP-1 PMs dropped significantly to 36.56%, whereas cells treated with PAP-1 and PAP-1 + MDRi maintained relatively higher viabilities of 80.26% and 73.48%, respectively. Similar trends were observed in B16F10 cells, with PAP-1 PMs exhibiting the highest cytotoxicity among all tested formulations, especially at higher concentrations. At 20 µmol/L, the viabilities of B16F10 cells treated with PAP-1, PAP-1 + MDRi, and PAP-1 PMs were 78.09%,76.71%, and 63.86%, respectively. At 50 µmol/L, the viabilities of B16F10 cells treated with PAP-1 PMs dropped to 16.7%, in contrast to 65.32% and 50.86% for PAP-1 and PAP-1 + MDRi treatments, respectively. In general, while PAP-1 and its combination with MDRi effectively reduced tumor cell viability, PAP-1 PMs demonstrated superior cytotoxicity among all the PAP-1 treatment formulations. This superiority is potentially attributed to the encapsulation of PAP-1 in PMs, which not only enhances the solubility of PAP-1 but also improves its internalization by tumor cells. As depicted in Figure 4, the blank polymers showed high cell viability in both cell lines. Specifically, Jurkat cells’ viability remained unaffected even at the maximum polymer concentration, whereas B16F10 showed a slight decrease to 84.88% viability at the highest polymer concentration. In addition, CSH and probenecid were nontoxic to both Jurkat and B16F10 cells at the tested concentrations (data not shown).

### 3.7. Apoptotic study

**Figure 5.**
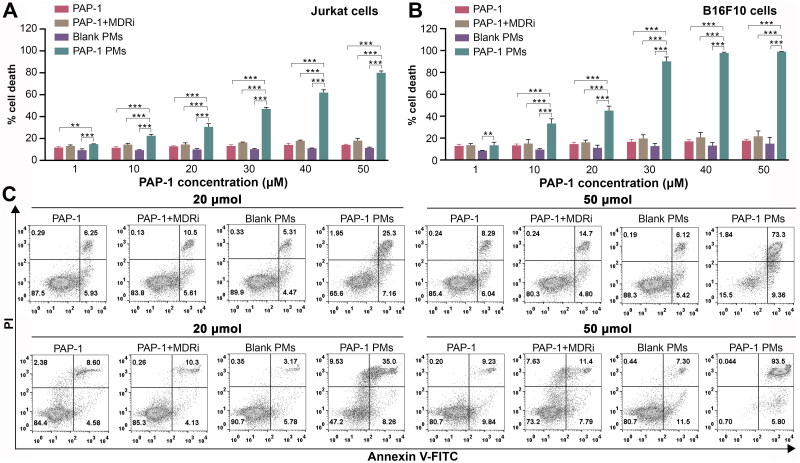
Apoptotic-inducing effect of different PAP-1 formulations on jurkat cells and B16F10 cells. (A) Jurkat cell death was analyzed through FACS by employing double staining with Annexin V and PI, utilizing the identical treatments previously described for CCK-8 analysis on cells. Apoptotic cells were quantified by considering annexin-positive cells. (B). B16F10 cells underwent the identical treatments as jurkat cells, enduring the same conditions for 24 hours. (C) Representative scatter plots of jurkat cells (upper) and B16F10 cells (bottom) obtained through FACS analysis demonstrated the effects of various PAP-1 formulations at concentrations of 20 μmol/L and 50 μmol/L for the 24-hour treatment period. Cell death data were expressed as mean ± standard deviation (*n* = 4). (*) *p* < 0.05, (**) *p* < 0.01, (***) *p* < 0.001.

To confirm that the observed decrease in cell viability was a result of increased apoptosis, flow cytometry analysis was conducted using Annexin-V FITC and propidium iodide (PI) staining. Consistent with the CCK-8 assay results, PAP-1 PMs induced a remarkably stronger apoptotic effect compared to PAP-1 and PAP-1 combined with MDRi (Figure 5). In Jurkat cells, the percentage of annexin-V positive cells (indicating apoptotic cells) was 12.82% in the PAP-1 group, 14.56% in the PAP-1 combined with MDRi group, and notably higher at 30.67% in the PAP-1 PMs group at a concentration of 20 µmol/L. As the drug concentration increased to 50 µmol/L, the apoptotic effect became more pronounced, with annexin-V positive cell percentages increasing to 14.16%, 18.08%, and a remarkable 80.02% for the PAP-1, PAP-1 combined with MDRi, and PAP-1 PMs groups, respectively. In B16F10 cells, similar apoptosis-inducing trends were observed across treatment groups. Notably, at a concentration of 50 µmol/L, the apoptotic cell percentages of B16F10 treated by PAP-1 or the PAP-1 combined with MDRi were 17.67% and 21.62%, respectively. In contrast, the PAP-1-loaded micelle treatment group exhibited a significantly more pronounced apoptosis-inducing effect, resulting in 98.92% of B16F10 cells undergoing apoptosis. The blank polymer micelles showed very low cytotoxicity for both the Jurkat and B16F10 cells. MDRi components, including CSH and probenecid, exhibited nontoxic effects on both Jurkat and B16F10 cells at the test concentrations (data not shown).

### 3.8. Caspase-3 activity assay

**Figure 6.**
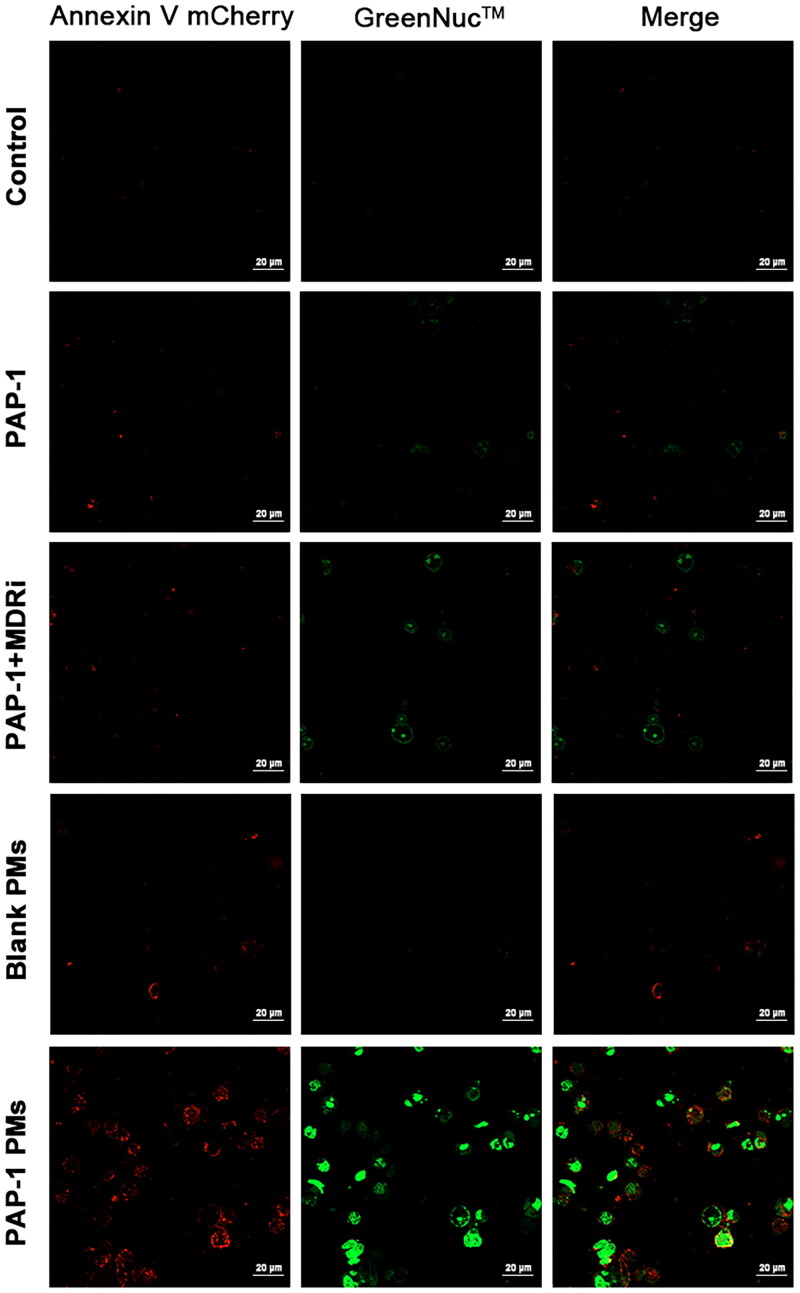
CLSM images of immunofluorescence, depicting caspase-3 activity and the effect apoptosis on jurkat cells exposed to various PAP-1 formulations at a concentration of 50 μmol/L for 24 hours. The cells were stained with GreenNuc^™^ caspase-3 and Annexin V-mCherry fluorescent probes. Images were captured using a laser confocal microscope (40×). The presence of red fluorescence from mCherry indicated cell apoptosis, while green fluorescence from GreenNuc^™^ signified increased caspase-3 activity in the cells.

Caspase-3, a crucial executor of cell apoptosis within the caspase signaling pathway, can be activated via mitochondrial apoptotic mechanisms. To investigate the involvements of caspases-3 in apoptosis triggered by various formulations, caspase-3/Annexin V dual staining assays were conducted (Figure 6). Activation of Caspase-3 was visualized using GreenNucTM caspase-3 substrate, a fluorescent polypeptide recognized by caspase 3/7 serving as the fluorogenic indicator, and simultaneously, phosphatidylserine externalization was detected via Annexin V-mCherry staining. Our findings indicated that PAP-1 and PAP1 + MDRi treatments caused a slight elevation in caspase-3 activity, accompanied by phosphatidylserine externalization. Remarkably, cells treated with PAP-1-loaded PMs showed significant enhancement in caspase-3 activity, which coincided with the occurrence of phosphatidylserine externalization, a hallmark of apoptosis.

### 3.9. JC-1

**Figure 7.**
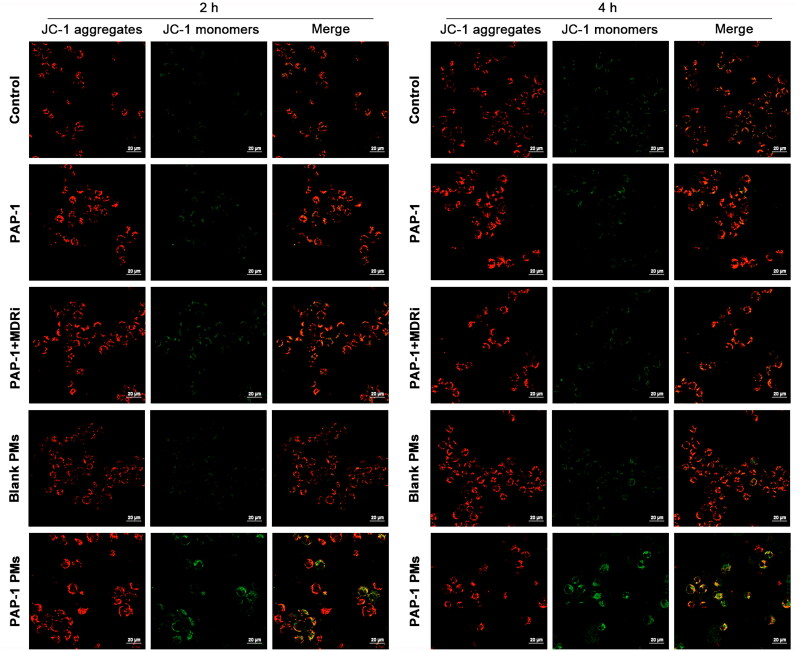
Detection of JC-1 signals in jurkat cells, which were treated with diverse PAP-1 formulations at a concentration of 50 μmol/L for durations of 2 or 4 hours, subsequently stained with the JC-1 fluorescent probe. Images captured by a laser confocal microscope at 40× magnification revealed red fluorescence emitted by JC-1 aggregates, which denoted a high mitochondrial membrane potential, whereas green fluorescence originating from JC-1 monomers was indicative of a reduction in mitochondrial membrane potential.

To further understand the apoptosis mechanism, we employed JC-1 staining to assess the impact of different treatments on mitochondrial membrane potential (Figure 7). JC-1 dye accumulates in healthy mitochondria due to the electrochemical gradient (ΔΨ_m_), emitting red fluorescence when aggregated, and green fluorescence in its monomeric cytosolic form. ΔΨ_m_ disruptions are evident through reduced red and increased green fluorescence. Our results, shown in Figure 7, revealed that PAP-1, PAP-1 combined with MDRi treatment, and PAP-1 PMs treatment decreased mitochondrial membrane potential. However, the PAP-1 PMs group exhibited a more significant mitochondrial membrane potential drop compared to the PAP-1 group and the PAP-1 and MDRi combination group. Conversely, exposure to blank PMs failed to significantly induce mitochondrial depolarization.

### 3.10. In vivo anti-tumor activity

**Figure 8.**
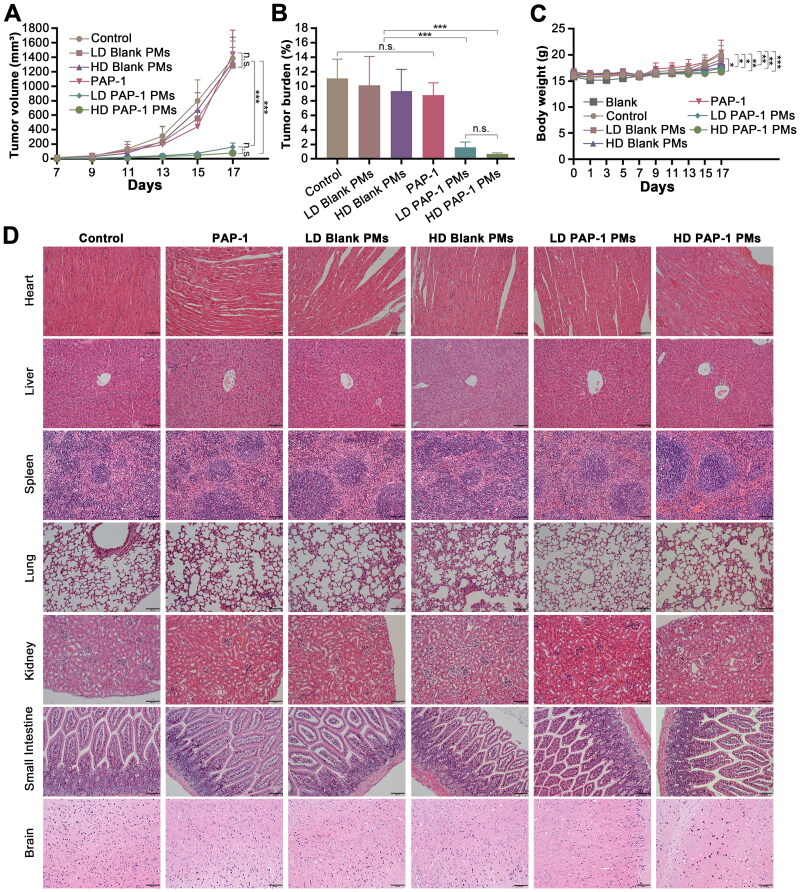
Antitumor effects of diverse PAP-1 formulations in B16F10 melanoma mouse model. (A) Tumor growth curves of mice treated with different formulations. (B) Tumor burden. (C) Body weight changes. The data were presented as mean ± standard deviation, with a sample size of six (*n* = 6). Statistical significance was denoted as non-significant (n.s.) for *p* > 0.05, (*) *p* < 0.05, (**) *p* < 0.01, (***) *p* < 0.001. (D). Hematoxylin/eosin staining from the heart, liver, spleen, lung, kidney, small intestine, and brain in the groups treated by different PAP-1 formulations.

The anti-tumor effect of PAP-1 PMs was investigated using C57BL/6 mice bearing the B16F10 melanoma (Figure 8). The changes in tumor volume of mice treated with various formulations from the first administration were depicted in Figure 8(A). Our results indicated that there was no significant difference between the control group, the PAP-1 treatment group (administered at 7 μg/g body weight), and the low-dose and high-dose blank micelles group. The results were consistent with findings reported by Leanza *et al.* that PAP-1, despite being the most potent and selective Kv1.3 inhibitor, failed to affect tumor size *in vivo*, likely due to its limited solubility in aqueous media. In comparison, PAP-1 PM treatment exhibited remarkable tumor suppression, reducing tumor volume by 88.23% and 94.26% in the low and high-dose groups, respectively. Although the high-dose group showed higher tumor inhibition rates, the difference was not statistically significant. Consistent with the above results, mice treated with PAP-1 PMs showed a remarkable reduction in tumor burden compared to those treated by PAP-1 alone, which indicated that the PAP-1 micelle system significantly enhanced the anti-tumor potency of PAP-1.

During the experiment, no obvious abnormalities were observed in the appearance, feeding behavior, or activity state of the mice. In terms of body weight, there was no significant difference among the untreated control (saline) mouse melanoma model group, the PAP-1 administration group, and the Blank PMs administration group. However, the body weight of mice in the control group and the PAP-1 administration group was larger than that of the blank group and PAP-1 PMs treatment group. This may be attributed to the fact that both the control group and the PAP-1 single administration group were unable to inhibit the growth of melanoma, leading to faster tumor growth in the later stages and subsequently causing an overall increase in the body weight of the mice. Also, these findings suggest that the administration of PAP-1 encapsulated in polymeric micelles (PAP-1 PMs) suppressed melanoma growth in mice, preventing the increase in body weight due to rapid tumor expansion. Additionally, the comparison between the PAP-1 PMs administration group and the blank group revealed no statistically significant difference in body weight, suggesting the safety profile of PAP-1 PMs administration. Furthermore, the H&E staining results explained that none of the tested formulations caused discernible damage to the main organs, including the heart, kidney, lung, spleen, liver, small intestine, and brain, as displayed in Figure 8(D). In general, all the results demonstrated that the PAP-1 PMs exerted notable anti-tumor effects but negligible toxicity.

### 3.11. Biodistribution

**Figure 9.**
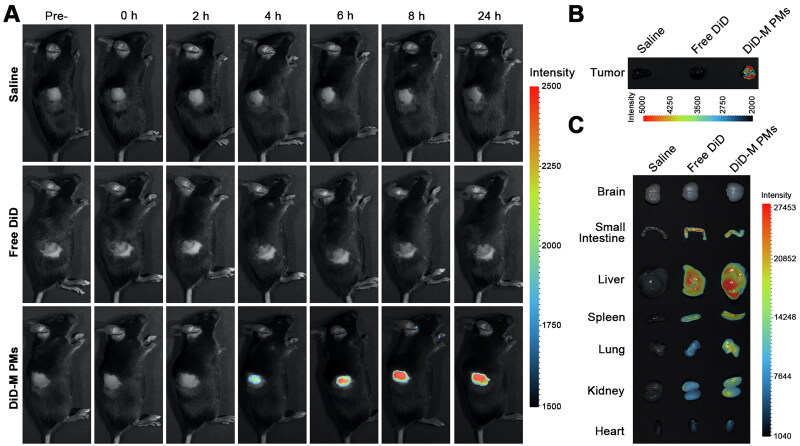
*In Vivo* DiD-marked micelles distribution in B16F10 melanoma tumor-bearing mouse models. (A) Representative photographs of live animals captured at pre-administration and at various time points post-administration (0, 2, 4, 6, 8, and 24 hours) for comparison. The images included the control group treated with saline, as well as groups receiving free DiD or DiD-marked micelles using IVIS^®^. (B). *Ex vivo* fluorescent images of the tumors and (C). main organs, including the brain, small intestine, liver, spleen, lung, kidney, and heart of mice after administration of PBS, free DiD, or DiD-marked micelles for 24 hours (*n* = 3).

The IVIS spectrum was used to investigate the biodistribution of DiD and DiD-marked micelles in mice bearing B16F10 melanoma (Figure 9). Pronounced fluorescence accumulation was observed in the tumors of mice administered with DiD-marked micelles, starting at 4 hours post-administration and gradually intensifying throughout the 24-hour observation period. This accumulation can be attributed to the enhanced permeability and retention (EPR) effect coupled with pH-sensitive controlled release behavior. In contrast, mice receiving free DiD exhibited minimal fluorescence accumulation in their tumors. To further evaluate the distribution patterns, mice were euthanized 24 hours after injection, and their tumors and key organs (brain, small intestine, liver, spleen, lung, kidney, and heart) were dissected for *ex-vivo* imaging. The fluorescent intensity detected in the tumors of the DiD-marked micelle treatment group was significantly higher than that of the DiD group. Additionally, strong fluorescence intensity was observed in metabolically active organs, particularly in the liver and kidney, for both the free DiD treatment group and the DiD-marked micelle treatment group, a common and expected outcome when using small-molecule probes. As a control, mice injected with saline showed no fluorescence in their tumors over the 24 hours. The mice were also sacrificed after a 24-hour period, and subsequent imaging of their tumors and organs confirmed the absence of fluorescence without the presence of DiD.

## 4. Conclusion

In this study, we encapsulated PAP-1 into mPEG-PAE, forming pH-sensitive polymeric micelles, aiming to improve PAP-1’s solubility, enhance tumor targeting, and potentially increase resistance to efflux caused by multidrug resistance (MDR) pumps, thereby enhancing PAP-1’s antitumor efficacy. The micelles self-assembled into nanostructures with a high entrapment efficiency of 91.35% and exhibited pH sensitivity. The release of PAP-1 from these micelles was significantly accelerated at lower pH, indicating pH-controlled drug release. Molecular simulations further supported that protonation of the tertiary amine groups of PAE influenced the self-assembly process. Compared with PAP-1 and PAP-1 PMs significantly enhanced cytotoxic and apoptosis induction. *In vivo*, PAP-1 PMs reduced tumor volume by 94.26% in a melanoma model, where free PAP-1 proved ineffective. Furthermore, encapsulation in micelles enhanced PAP-1’s tumor accumulation. *In vivo* experiments in mice confirmed that PAP-1 PMs treatment had not shown significant toxicity or side effects, although we are currently evaluating the long-term toxicological effects of PAP-1 PMs. Overall, these PAP-1-loaded pH-responsive polymeric micelles may be an efficient antitumor drug delivery system, paving the way for the design of advanced nanomedicines in cancer chemotherapy. Our future research will investigate whether PAP-1-loaded polymeric micelles can further enhance tumor suppression by mechanisms beyond improved solubility, such as by promoting drug endocytosis and mitigating P-gp pump-mediated efflux, driven by their superior in vivo and in vitro tumor suppression observed.

## Supplementary Material

Figure_S2.jpg

Figure_S1.tif

Ethical Reliable High_PAP1 loaded Polymeric Micelles.pdf

TableS1.xlsx

Ethical Approval Letter.pdf

## Data Availability

The data that support the findings of this study are available on request from the corresponding author.
