# Supplementary figures and images for: Reliable high-PAP-1-loaded polymeric micelles for cancer therapy: preparation, characterization, and evaluation of anti-tumor efficacy
Source: Drug Deliv. 2025 Apr 10;32(1):2490269. doi: 10.1080/10717544.2025.2490269 (PMC11986873; doi:10.1080/10717544.2025.2490269)

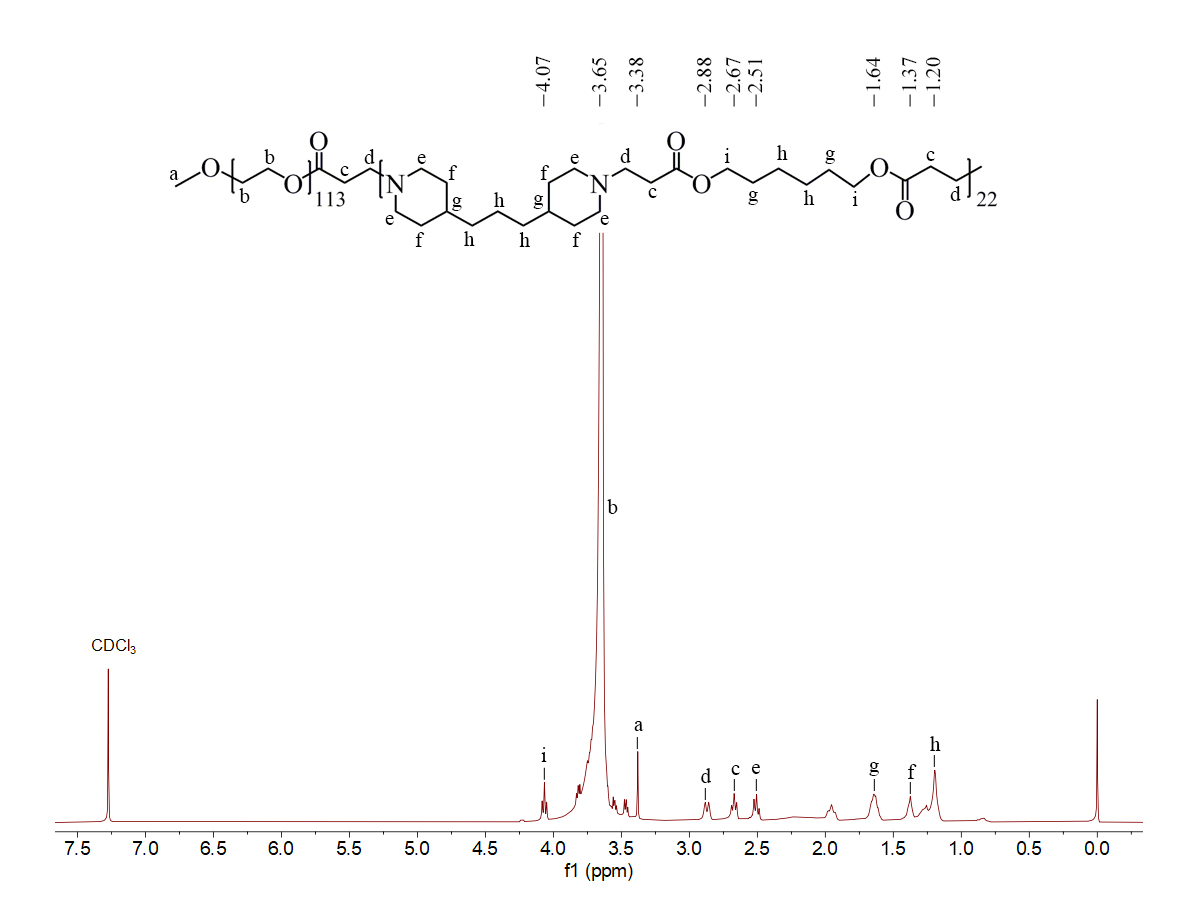

Supplement: Figure_S2.jpg [file IDRD_A_2490269_SM9418.jpg]

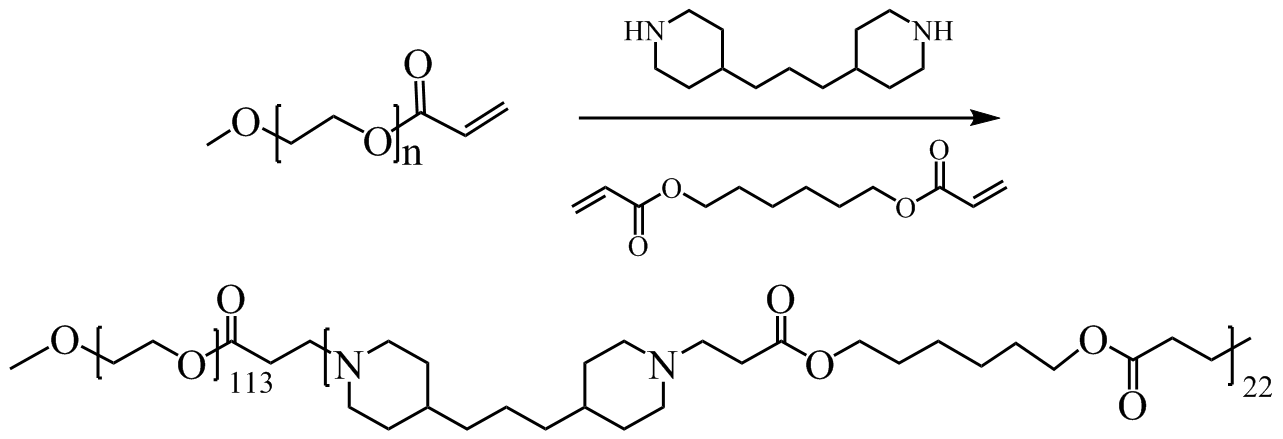

Supplement: Figure_S1.tif [file IDRD_A_2490269_SM9417.tif]
