# Supplementary material for: Reliable high-PAP-1-loaded polymeric micelles for cancer therapy: preparation, characterization, and evaluation of anti-tumor efficacy
Source: Drug Deliv. 2025 Apr 10;32(1):2490269. doi: 10.1080/10717544.2025.2490269 (PMC11986873; doi:10.1080/10717544.2025.2490269)
Supplement: Ethical Reliable High_PAP1 loaded Polymeric Micelles.pdf [file IDRD_A_2490269_SM9416.pdf]

Table for Animal Experimental Ethical Inspection No.: 202011014

<table>
<tr><td rowspan="11">Concerned information written by applicant</td><td colspan="3">Applicant： Ye, Fang</td></tr>
<tr><td colspan="3">Education of applicant：Ph.D. Professional title：Assistant Professor</td></tr>
<tr><td colspan="3">Study title： Reliable High-PAP-1-loaded Polymeric Micelles for cancer therapy: Preparation, Characterization, and Evaluation of Anti-Tumor Efficacy</td></tr>
<tr><td colspan="3">Aim of experiment： The aim of the experiments was to evaluate the anti-tumor activities of PAP-1-loaded polymeric micelles in vivo using a C57BL/6 mouse model bearing B16F10 tumors, and to investigate the biodistribution of the micelles through fluorescence imaging.</td></tr>
<tr><td colspan="3">Fund sources：This work was supported by the National Natural Science Foundation of China (Grant number 81760555 and 32060233), the Natural Science Foundation of Guangxi Province (Grant number 2017GXNSFAA198049).</td></tr>
<tr><td rowspan="4">Animal Information</td><td colspan="2">Source of animal：the Experimental Animal Center of Guangxi Medical University</td></tr>
<tr><td colspan="2">Species or strain：C57BL/6J Grade：SPF Specifications：4 weeks</td></tr>
<tr><td>Number： 76 ♀： 76 ♂：</td><td>Application date： 11/1/2020</td></tr>
<tr><td>Entering date： 03/1/2021</td><td>Ending date： 08/01/2023</td></tr>
<tr><td colspan="3">1.Outline of experiments； experimental methods；observational index：
Outline of Experiment:
To evaluate the anti-tumor activities of different treatments in vivo, an orthotopic mouse B16F10 melanoma model was established. B16F10 cells were cultured, harvested, and injected into the right flanks of C57BL/6 mice. The experimental mice were then randomly divided into six groups and administered various treatments, including a control group (saline), free PAP-1 at 7 μg/g body weight, low and high doses of blank polymeric micelles (PMs), and low and high doses of PAP-1-loaded PMs. Treatments were administered intraperitoneally on days 5, 7, 9, and 11 post-tumor inoculation. Tumor volumes were monitored by measuring tumor dimensions using calipers and calculating the volume with the formula $V = (L\times W^2)/2$. After 17 days, tumors were excised and weighed to determine the tumor burden. Additionally, major organs were dissected from the sacrificed mice to assess potential toxicity or side effects of the treatments.
Experiment Methods:
5×10^4 B16F10 cells were subcutaneously injected into the right flanks of C57BL/6 mice. After the tumor inoculation, all experimental mice were randomly divided into six groups (n = 6) and administered the following treatments: a control group receiving saline, a group receiving free PAP-1 at a dose of 7 μg/g body weight, two groups receiving blank polymeric micelles (PMs) at low and high doses, and two groups receiving PAP-1-loaded PMs at low and high doses equivalent to 7 μg/g and 14 μg/g of PAP-1, respectively. The formulations were administered intraperitoneally on the 5th day after tumor inoculation, followed by subsequent administrations on days 7, 9, and 11. During the experiment, tumor volumes and body weights were carefully monitored and recorded. Tumor dimensions, specifically lengths (L) and widths (W), were measured using calipers, and tumor volumes were calculated using the formula V = (L×W2)/2, where W is the shorter dimension compared to L. After 17 days, the tumors were excised, and their weights were determined to calculate the tumor burden using the formula: tumor burden (%) = $(W_{tumor}/W_{mice}) \times 100$.
Additionally, at the end of the experiment, main organs such as the heart, kidney, lung, spleen, liver, small intestine,</td></tr>
</table>

and brain were dissected from the sacrificed mice for further analysis.

Observational Index:

The primary observational index in this experiment was the tumor volume, which was measured using calipers and calculated based on the dimensions of the tumor. Body weights were also recorded as an indicator of the mice's overall health and potential toxicities. Tumor burden was determined by excising and weighing the tumors, providing a quantitative measure of the tumor growth. Finally, the dissection of major organs allowed for the inspection of potential toxicity or side effects of the treatments.

2.Humane endpoint or experimental terminative indicator

The tumor size in mice should not exceed 2000mm^3, or the animal should be euthanized.

3.Executing animal method:

Cervical dislocation and carbon dioxide inhalation.

4.Major measure for 3Rs:

For the Reduction aspect, we aimed to minimize the number of animals used by carefully planning the experiment and ensuring maximum utilization of each mouse. By randomizing the allocation of mice into treatment groups, we aimed to reduce bias and ensure that the results would be representative of the entire population. For Replacement, we utilized the mouse model as a necessary alternative to human experimentation. However, we also explored the potential for other in vitro that could potentially replicate the tumor environment more accurately, while minimizing the use of animals. In terms of Refinement, tumor volumes and body weights were monitored regularly to assess the animals' general health and well-being. Additionally, the use of intraperitoneal administration aimed to minimize stress and discomfort compared to other routes of administration. The organs dissected from the sacrificed mice were also handled with care to ensure minimal tissue damage. By adhering to these principles of the 3Rs, we aimed to ensure that our research was conducted in an ethical and humane manner, while still achieving the necessary scientific objectives.

Signature of applicant: 叶芳　　Telephone: 15676120104

Results of inspection	Study director: Agree ☐ Disagree ☐ Signature
	Opinion from laboratory animal facility:<br>Agree ☑ Disagree ☐<br>欧阳铁强<br>Signature:

	The Animal Care & Welfare Committee：<br><br>Agree ☑ Disagree ☐<br><br>广西医科大学实验动物伦理委员会<br>Stamp<br>4501030257945
Supplement：<br><br>First trial ☑ reexamine No.	

Notes : Animal ethics review follows the *Guiding Opinions on the Treatment of Laboratory Animals* issued by the Ministry of Science and Technology of the People's Republic of China and the *Laboratory Animal-Guideline for Ethical Review of Animal Welfare* issued by the National Standard GB/T35892-2018 of the People's Republic of China. If there are any uncertainties, please refer to these two documents or consult The Animal Care & Welfare Committee of Guangxi Medical University.
