# Supplementary material for: Reliable high-PAP-1-loaded polymeric micelles for cancer therapy: preparation, characterization, and evaluation of anti-tumor efficacy
Source: Drug Deliv. 2025 Apr 10;32(1):2490269. doi: 10.1080/10717544.2025.2490269 (PMC11986873; doi:10.1080/10717544.2025.2490269)
Supplement: Ethical Approval Letter.pdf [file IDRD_A_2490269_SM9414.pdf]

Approval for Animal Ethics Application - Ref#: 202011014

This letter serves to certify that the application submitted by Ye, Fang, for the study titled "Reliable High-PAP-1-loaded Polymeric Micelles for Cancer Therapy: Preparation, Characterization, and Evaluation of Anti-Tumor Efficacy", with reference number 202011014, has been approved.

The application for animal ethics related to the aforementioned research was thoroughly reviewed by The Animal Care & Welfare Committee of Guangxi Medical University. The review was conducted in compliance with the Guiding Opinions on the Treatment of Laboratory Animals issued by the Ministry of Science and Technology of the People's Republic of China, as well as the Laboratory Animal-Guideline for Ethical Review of Animal Welfare issued by the National Standard GB/T35892-2018 of the People's Republic of China.

Best regards,
The Animal Care & Welfare Committee of Guangxi Medical University

Ouyang Yiqiang

广西医科大学实验动物伦理委员会
